# Patient fibroblasts-derived induced neurons demonstrate autonomous neuronal defects in adult-onset Krabbe disease

**DOI:** 10.18632/oncotarget.12812

**Published:** 2016-10-21

**Authors:** Su Min Lim, Byung-Ok Choi, Seong-il Oh, Won Jun Choi, Ki-Wook Oh, Minyeop Nahm, Yuanchao Xue, Jae Hyeok Choi, Ji Young Choi, Young-Eun Kim, Ki Wha Chung, Xiang-Dong Fu, Chang-Seok Ki, Seung Hyun Kim

**Affiliations:** ^1^ Biomedical Research Institute, Hanyang University, Seoul, Republic of Korea; ^2^ Cell Therapy Center, Hanyang University Hospital, Seoul, Republic of Korea; ^3^ Department of Neurology and Neuroscience Center, Samsung Medical Center, Sungkyunkwan University School of Medicine, Seoul, Republic of Korea; ^4^ Department of Neurology, Busan Paik Hospital, Inje University College of Medicine, Busan, Republic of Korea; ^5^ Department of Neurology, Sheikh Khalifa Specialty Hospital, Ras Al Khaimah, United Arab Emirates; ^6^ Department of Neurology, College of Medicine, Hanyang University, Seoul, Republic of Korea; ^7^ Key Laboratory of RNA Biology, Institute of Biophysics, Chinese Academy of Sciences, Beijing, China; ^8^ Green Cross Genome, Yongin, Republic of Korea; ^9^ Department of Biological Sciences, Gongju National University, Gongju, Republic of Korea; ^10^ Department of Cellular Molecular Medicine, University of California, San Diego, La Jolla, CA, USA; ^11^ Department of Laboratory Medicine and Genetics, Samsung Medical Center, Sungkyunkwan University School of Medicine, Seoul, Republic of Korea

**Keywords:** krabbe disease, globoid cell leukodystrophy, β-galactosylceramidase, psychosine, induced neuron, Gerotarget

## Abstract

Krabbe disease (KD) is an autosomal recessive neurodegenerative disorder caused by defective β-galactosylceramidase (GALC), a lysosomal enzyme responsible for cleavage of several key substrates including psychosine. Accumulation of psychosine to the cytotoxic levels in KD patients is thought to cause dysfunctions in myelinating glial cells based on a comprehensive study of demyelination in KD. However, recent evidence suggests myelin-independent neuronal death in the murine model of KD, thus indicating defective GALC in neurons as an autonomous mechanism for neuronal cell death in KD. These observations prompted us to generate induced neurons (iNeurons) from two adult-onset KD patients carrying compound heterozygous mutations (p.[K563*];[L634S]) and (p.[N228_S232delinsTP];[G286D]) to determine the direct contribution of autonomous neuronal toxicity to KD. Here we report that directly converted KD iNeurons showed not only diminished GALC activity and increased psychosine levels, as expected, but also neurite fragmentation and abnormal neuritic branching. The lysosomal-associated membrane proteins 1 (LAMP1) was expressed at higher levels than controls, LAMP1-positive vesicles were significantly enlarged and fragmented, and mitochondrial morphology and its function were altered in KD iNeurons. Strikingly, we demonstrated that psychosine was sufficient to induce neurite defects, mitochondrial fragmentation, and lysosomal alterations in iNeurons derived in healthy individuals, thus establishing the causal effect of the cytotoxic GALC substrate in KD and the autonomous neuronal toxicity in KD pathology.

## INTRODUCTION

Krabbe disease (KD) characterized by globoid cell leukodystrophy is an autosomal recessive lysosomal storage disease caused by mutations in the β-galactosylceramidase (*GALC*) gene that impair the enzymatic function. While frequently detected in infants, KD has also been found as an adult-onset disease [1, 2]. Compared to rapid clinical progression in infants, adult-onset KD patients show less severe phenotype with slower disease progression [2]. In most enzymatic lysosomal diseases, deficiencies in lysosomal enzyme activities are correlated with the degree of clinical severity. However, such relationship appears obscure with KD patients [3, 4]. This discrepancy between loss of enzymatic activity and disease phenotype has made it difficult to predict the phenotype of *GALC* mutations, which are often heterozygous in KD patients.

During myelin turnover, GALC catabolizes the primary substrate galactosylceramide (GalCer) to galactose and ceramide, and the secondary substrate psychosine to galactose and sphingosine [5]. Both GalCer and psychosine are processed in the lysosome and their recycled components have been found to enter the remyelination pathway in the nervous system [6]. This leads to the proposal that compromised GALC enzymatic activity in KD results in insufficient degradation of both GalCer and psychosine, thus causing reduced remyelination efficiency in the nervous system [1].

While impaired remyelination has been thought to be a direct cause of axonal dystrophy in KD, recent evidence suggests that myelin loss appears to be insufficient to count for defects in neurons and axons in the Twitcher mouse model, because cultured neurons from the Twitcher mice exhibit typical neuronal and axonal defects in the absence of disrupted myelinating glia, thus indicating autonomous neuronal damage in KD [7–9]. Moreover, psychosine has also been found to alter the angiogenesis process in the murine model, and linked to neuronal inclusion of misfolded and aggregated α-synuclein in postmortem brains from both infantile and late onset KD patients [10, 11]. These studies all point to potential autonomous neuronal dysfunction independent of myelin defects in leukodystrophic pathology, which may in fact precede myelin loss. However, our understanding of the pathogenic role of myelin-independent neuronal degeneration in KD has been hampered by the lack of patient-derived cellular models that are able to recapitulate human KD pathologies.

In the present study, we generated and characterized induced neurons (iNeurons) derived from two adult-onset KD patients carrying *GALC* (p.[K563*];[L634S]) and (p.[N228_S232delinsTP];[G286D]). Using these disease-relevant and the patient-specific cell models, we report abnormal GALC enzymatic activities and psychosine levels in patient cells. In patient-derived iNeurons, we demonstrate a direct relationship of *GALC* mutation and abnormal psychosine accumulation with axonal and dendritic defects with morphological and functional impairments in lysosomes and mitochondria. These myelin-independent axonal and neuronal defects thus strongly argue for autonomous neuronal toxicity in adult-onset KD.

## RESULTS

### Clinical manifestations of two unrelated adult-onset KD patients

**Figure 1:**
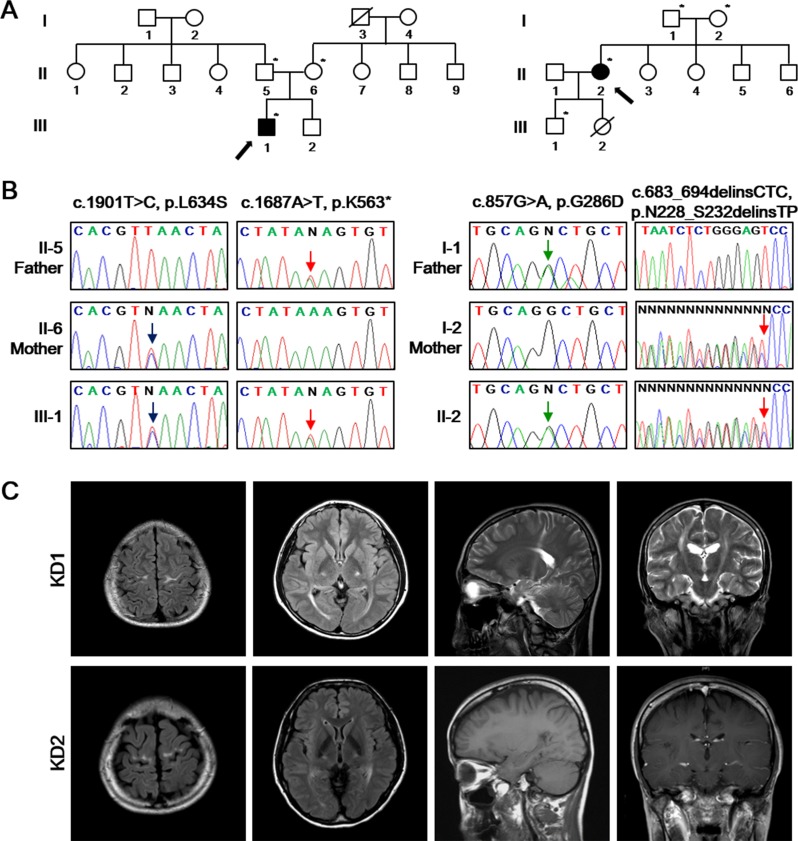
Identification of the disease-causing mutations of *GALC* in two unrelated families **A.** Pedigrees of two KD patients (left: KD1; right: KD2) with ***GALC*** mutations. The available DNA samples are indicated by asterisks (*). The probands are marked with arrows with filled symbols. Hashed symbols indicate deceased individuals. **B.** Sanger sequencing of the *GALC* gene (Reference mRNA sequence: NM_000153.3) from the patients identified c.1901T>C (p.L634S) combined with c.1687A>T (p.K563*) (left) and c.857G>A (p.G286D) combined with c.683_694delinsCTC (p.N228_S232delinsTP) (right). **C.** Brain MR images of KD1 patient revealed the severe high intensity signal of the precentral gyrus, corona radiata, posterior limb of internal capsule, cerebral peduncle of the bilateral pyramidal tracts and optic radiation on fluid-attenuated inversion recovery (FLAIR) and T2-weighted images. Brain MRI of the patient KD2 revealed the high intensity signal in the precentral gyrus and posterior limb of internal capsule, without signal abnormality on the T1-weighted and gadolinium-enhanced T1-weighted images.

A 12-year-old male (KD1) has been suffering slow progressive spastic gait disturbance since three months ago. He had one younger brother and their parents were non-consanguineous. In the family history, his maternal grandfather (I-3) died at age 40 from an unknown cause of cardiac arrest, but other family members including his parents (II-5 and 6) and brother (III-2) remained healthy at the time of this analysis (Figure 1A, left). Neurological examination of the patient revealed mild spastic weakness on lower extremities, exaggerated patellar tendon reflexes, and positive Babinski reflex and ankle clonus. Postural tremors with mild dysmetria in finger-to-nose test were also noted in his upper limbs. While there was no detectable defect in general developmental state, sensory function, and autonomic function, the patient showed abnormal phonemic generative naming ability (below 1% of age group) in comprehensive neuropsychological tests, suggesting frontal dysfunction. Laboratory studies of biochemical and cerebrospinal fluid screening indicated that plasma electrolytes, liver function, calcium, phosphate, thyroid function, full blood count, vitamin B-12 and folate, syphilis serology, and autoantibody profile, were all unremarkable. Highly specialized laboratory analyses further excluded some rare metabolic disorders and the level of very long chain fatty acids (VLCFA) level remained in a normal range. The enzymatic activity of hexosaminidase A and arylsulfatase A were also within the reference range. Importantly, the GALC enzymatic activity detected by LC-MS/MS in leukocytes was markedly decreased (1.8 nmol/hr/mg protein), in comparison to the activity that reached 137.5 nmol/hr/mg protein in an age-matched control (Table 1).

**Table 1:** Patients and controls whose skin fibroblasts were studied

Characteristic	KD1	KD2	CTL1	CTL2	CTL3
Sex	M	F	F	F	M
*GALC* genotype	c.[1687A>T];[1901T>C] p.[K563*];[L634S]	c.[683_694delinsCTC];[857G>A] p.[N228_S232delinsTP];[G286D]	N/A	N/A	N/A
Phenotype	Adult onset	Adult onset	Asymptomatic	Asymptomatic	Asymptomatic
Age at onset, yr	12	30	N/A	N/A	N/A
Age at biopsy, yr	12	38	15	35	58
Enzyme activity (nmol/hr/mg protein) ^a^	1.8	5.1	N/A	N/A	N/A

Enzyme activity indicates β-galactosylceramidase activity in lymphocytes.

CTL = control; F = Female; KD = Krabbe disease; M = male; N/A = not applicable

a Healthy control enzyme activity = 50-150 nmol/hr/mg protein.

Direct sequencing of the *GALC* gene (Reference mRNA sequence: NM_000153.3) in this KD1 patient identified a novel nonsense mutation (c.1687A>T:p.K563*) in exon 15 in combination with a known missense mutation (c.1901T>C:p.L634S) in exon 16 (Figure 1B, left). Because his parents did not show any abnormal phenotype, we suggest that such compound heterozygous mutations are likely responsible for various disease phenotype in the patient. Brain MR images further revealed high-intensity lesions in the precentral motor cortex, corona radiata, posterior limb of the internal capsule, cerebral peduncle of the bilateral pyramidal tracts and optic radiation, with sparing of the periventricular white matter in fluid attenuated inversion recovery (FLAIR) and T2-weighted images (Figure 1C). Visual evoked potentials showed prolonged P100 latency in both eyes and prolonged central motor conduction times were observed in transcranial motor-evoked potentials.

We recently identified another adult-onset KD patient. This 38-year-old female (KD2) was the eldest daughter of healthy non-consanguineous parents. She was born at full term by spontaneous vaginal delivery and the perinatal history was unremarkable. At the age of 30, she started to complain progressive gait difficulty and spastic gait disturbance. The family members including her parents (I-1 and 2) and her younger brothers and sisters (II-3, 4, 5, and 6) remained healthy at the time of this analysis (Figure 1A, right). Neurological examination at the age of 38 revealed mild spastic weakness on lower extremities, exaggerated bilateral patellar tendon reflexes, ankle clonus and positive Babinski sign. She was able to walk independently harboring postural instability. Postural tremors with mild dysmetria in finger-to-nose test were also noted in her upper limbs.

The laboratory studies of biochemical and cerebrospinal fluid screenings showed normal activities. The level of very long chain fatty acids (VLCFA) level, and the enzymatic activity of hexosaminidase A and arylsulfatase A were within the reference range. The GALC enzymatic activity detected by LC-MS/MS in leukocytes was evidently decreased (5.1 nmol/hr/mg protein) compared to 72.9 nmol/hr/mg protein in an age-matched control. We also performed capillary sequencing of the *GALC* gene in this KD2 patient and identified the compound heterozygous mutations including c.857G>A (p.G286D) in exon 8, and c.683_694delinsCTC (p.N228_S232delinsTP) in exon 7 (Figure 1B, right). Brain MRI revealed high-intensity signal in the precentral gyrus and posterior limb of the internal capsule, but showed no signal abnormality on the T1-weighted and contrast-enhanced T1-weighted images (Figure 1C). Visual evoked potentials were prolonged P100 latency in both eyes. These features suggest that KD2 has a similar array of clinical symptoms to KD1, despite the fact they carry distinct mutations in their *GALC* genes.

### Subcellular localization of mutant GALC proteins in transfected cells and characterization of cultured skin fibroblasts from KD patients

In order to examine subcellular localization of GALC, we overexpressed the cDNA encoding wild-type or mutant GALC C-terminally tagged with FLAG or HA in HeLa cells. In cells expressing either FLAG-tagged wild-type, p.L634S, p.G286D, or HA-tagged p.K563*, p.N228_S232delinsTP GALC construct, all exogenous proteins were predominantly co-localized with the endoplasmic reticulum (ER) marker (Calnexin), but not with the lysosomal marker lysosomal-associated membrane proteins 1 (LAMP1) (Supplementary Figure 1). Therefore, the cellular localization of each overexpressed mutant GALC protein was indistinguishable from that of the wild-type enzyme.

**Figure 2:**
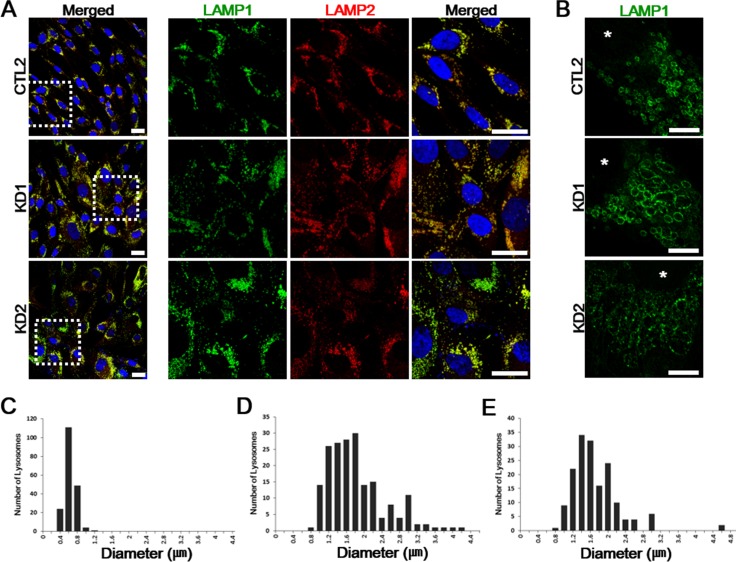
Immunofluorescent analysis of the fibroblast cultures from the KD patients **A.** Control and both KD1 and KD2 fibroblasts were fixed, permeabilized, and stained. LAMP1 (green) and LAMP2 (red) are both expressed higher in the patients compared to a representative healthy control fibroblasts. Enlarged images of the regions highlighted by dashed white squares in merged images (left) are in the right columns. DNA is identified by DAPI. Scale bars: 25 μm. **B.** Representative STED images of LAMP1 morphology differences in healthy controls and KD fibroblasts. LAMP1 morphologies are altered with enlarged and fragmented LAMP1-positive vesicles. Nuclei are indicated by asterisks (*). Scale bars: 5 μm. **C.**-**E.** The increased distribution ranges of lysosome diameters in patient fibroblasts (D, KD1: range, 0.74-4.15 μm; E, KD2: range, 0.75-4.68 μm) compared to healthy control fibroblasts (C, CTL: range, 0.36-1.03 μm). For each plot, *n* = 20 cells.

Patient-derived skin fibroblasts were previously used to characterize other lysosomal storage diseases, such as Niemann Pick type C or mucolipidosis type VI, both showing enlarged lysosomes [12]. To determine whether such morphological changes in lysosome were also related to diminished GALC activity in KD leukocytes, we cultured fibroblasts from healthy controls (CTL1, CTL2, CTL3) and two KD patients KD1 and KD2. Confocal imaging of both lysosomal-associated membrane protein 1 and 2 (LAMP1 and LAMP2) revealed increased LAMP1 and LAMP2-positive vesicles in both patient fibroblasts, compared to all healthy controls (Figure 2A). In order to determine potentially altered lysosomal morphology in the patient fibroblasts, we imaged the lysosomal membrane using super-resolution stimulated emission depletion (STED) microscopy. STED imaging of both patient fibroblasts revealed markedly enlarged polygonal lysosome structures of various sizes, while control fibroblast showed uniformed spherical puncta shaped lysosomes (Figure 2B). Alterations in vesicle size in the patient cells were further confirmed by comparing vesicle diameters between healthy controls and the patients (Figure 2C-2E). Together, these data suggest that lysosome enlargement and increased expression of LAMP1 and 2, both common features in other lysosomal storage diseases, were also observed in KD patient-derived fibroblasts.

### KD iNeurons directly converted from the patient skin fibroblasts demonstrates reduced GALC activity and accumulated of psychosine

**Figure 3:**
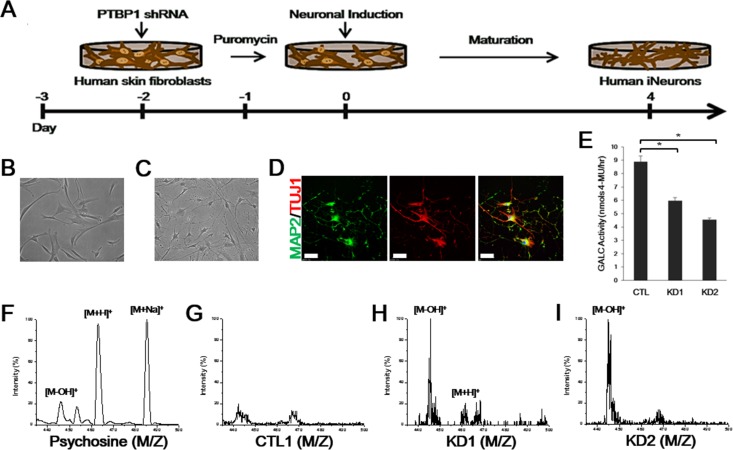
GALC enzymatic activity and MALDI-TOF mass spectrometry of iNeurons from KD patients **A.** Schematic representation of the conversion process from human fibroblasts to iNeurons in response to PTBP1 shRNA. Within one day of neuronal induction, cells underwent morphological changes from flat fibroblastic spindle-shaped cells **B.** to distinctive round cell bodies with neurites **C.** (B, C = 200×). **D.** Immunofluorescence of cultures at day 8 reveals iNeurons expressing neuronal markers, MAP2 (green, neuronal dendrite marker) and TUJ1 (red, the early neuronal marker βIII-tubulin). DNA is identified by DAPI. Scale bars: 100 μm. **E.** GALC activity toward MUGal substrate (nmols/hr) measured from 15 μg of whole-cell protein extracts from a representative healthy control and KD iNeurons. Activity is presented as means ± SEM, *n* = 4. One-way ANOVA followed by Tukey multiple comparisons test; *p < 0.001. **F.** The psychosine standard substance of the [M-OH]^+^ signal at mass/charge (m/z) 445, [M+H]^+^ at m/z 463, and [M+Na]^+^ at m/z 485 are analyzed by positive ion mode MALDI-TOF mass spectrometry. Compared to control iNeurons **G.**, distinctive molecular ion peaks [M-OH]^+^ at m/z 445 and [M+H]^+^ at m/z 462 could be detected in KD1 iNeurons **H.**. **I.** Strong peak of [M-OH]^+^ was also obtained in KD2 iNeurons.

Recent reports showed the association of myelin-free neuronal and axonal defects with the KD pathology in the Twitcher model [7, 8]. We therefore trans-differentiated skin fibroblasts from the KD patients and healthy controls (CTL1, CTL2, CTL3) into induced neurons (iNeurons) by using a lentivirus that represses PTBP1, an RNA binding protein whose down-regulation has been demonstrated to be necessary and sufficient to induce iNeurons from both murine and human fibroblasts [13, 14]. To promote iNeuron conversion, puromycin-resistant cells at 60 h post-infection, were replaced in N3 media containing the basic fibroblast growth factor (bFGF), supplemented with neurotrophic factors (Figure 3A). Within 1 day after infection, cells underwent morphological changes from flat spindle-shaped fibroblasts to spherical-shaped cell bodies with neurites (Figure 3B, 3C). The advanced maturated morphology of iNeurons with dendritic branching was confirmed by immunostaining with the neuronal dendrite marker MAP2 at day 8 of neuronal induction (Figure 3D). To test if compound heterozygous mutations in GALC affected the enzymatic activity in iNeurons, we measured the GALC activity from a representative control and both patient whole cell extracts using an enzymatic assay, as previously described [15]. GALC from both patient iNeurons showed significantly reduced GALC activity than that of controls (KD1 = ~67%; KD2 = ~51% of control iNeurons activity) (Figure 3E). MALDI-TOF mass spectrometry analysis of pure psychosine revealed ion peaks at m/z 445 ([M-OH]^+^), 463 ([M+H]^+^), and 485 ([M+Na]^+^) as expected in the previous study (Figure 3F) [16]. In control iNeurons, we detected no distinctive intensity of those peaks for psychosine (Figure 3G). MALDI-TOF analysis of patient iNeurons also revealed a strong ion peak at m/z 445 and a slight peak at m/z 462 for KD1 iNeurons, indicating increased psychosine levels in the patient cells (Figure 3H). Similarly, a strong intensity of a peak was also detected at m/z 445 in KD2 iNeurons (Figure 3I). Taken together, these data demonstrated that both adult-onset KD patient-derived iNeurons show significantly reduced GALC enzymatic activity along with increased psychosine levels, despite the patients carry distinct sets of mutations in the *GALC* gene, thus providing ideal cellular models to link the genotypes to other potential disease phenotypes.

### KD iNeurons demonstrate impaired lysosome morphology and accumulation

**Figure 4:**
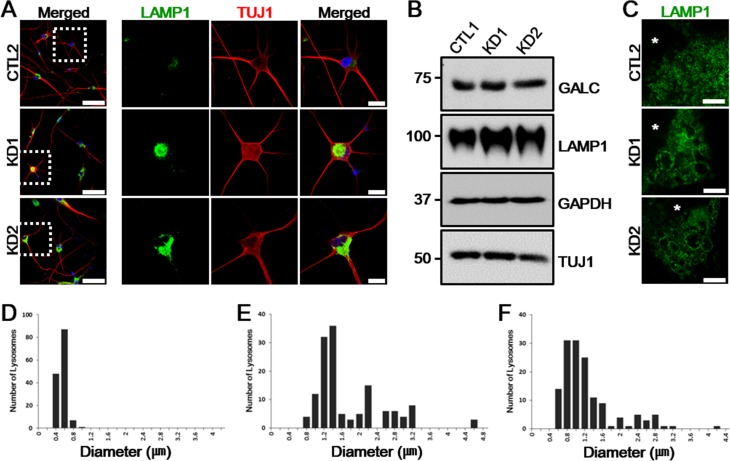
LAMP1 vesicles accumulate in KD iNeurons **A.** LAMP1 (green) expression is increased in patient iNeurons positive for TUJ1 (red). Enlarged images of the regions highlighted by dashed white squares in merged images (left) are in the right columns. DNA is identified by DAPI. Scale bars: 100 μm (enlarged images: 25 μm). **B.** Whole-cell protein extracts from a representative healthy control and patient iNeurons analyzed by immunoblotting show similar amount of GALC expression but the increase in LAMP1 was confirmed. GAPDH and TUJ1 are shown as loading controls (*n* = 4). **C.** Representative STED images of LAMP1 morphology differences in control and the two KD patient iNeurons. Alterations of LAMP1 morphologies are demonstrated with enlarged and irregularly shaped LAMP1-positive vesicles in KD iNeurons. Nuclei are indicated by asterisks (*). Scale bars: 5 μm. **D.**-**F.** The increased distribution ranges of lysosome diameters in KD iNeurons (E, KD1: range, 0.79-4.64 μm; F, KD2: range, 0.63-4.3 μm) compared to control iNeurons (D, CTL: range, 0.25-0.99 μm)). For each plot, *n* = 15 cells.

By both immunostaining and immunoblot analysis, we first characterized distinctive disease phenotypes on iNeurons. We found that the level of LAMP1 was increased in KD iNeurons, which was well coincided with the results obtained by the fibroblast modeling test (Figure 4A, 4B). To determine whether the morphology of lysosomal membrane was also altered in patient iNeurons, as in patient fibroblasts, we imaged the lysosomal membrane using STED microscopy. Indeed, STED imaging of patient iNeurons revealed that LAMP1-positive vesicles were enlarged and irregularly shaped compared to control iNeurons (Figure 4C). Alterations in vesicle size in patient iNeurons were further confirmed by comparing vesicle diameter ranges between control and patient iNeurons (Figure 4D-4F). Therefore, lysosomal impairment remained similarly manifested after converting patient fibroblasts to iNeurons.

### KD iNeurons reveal axonal defects

**Figure 5:**
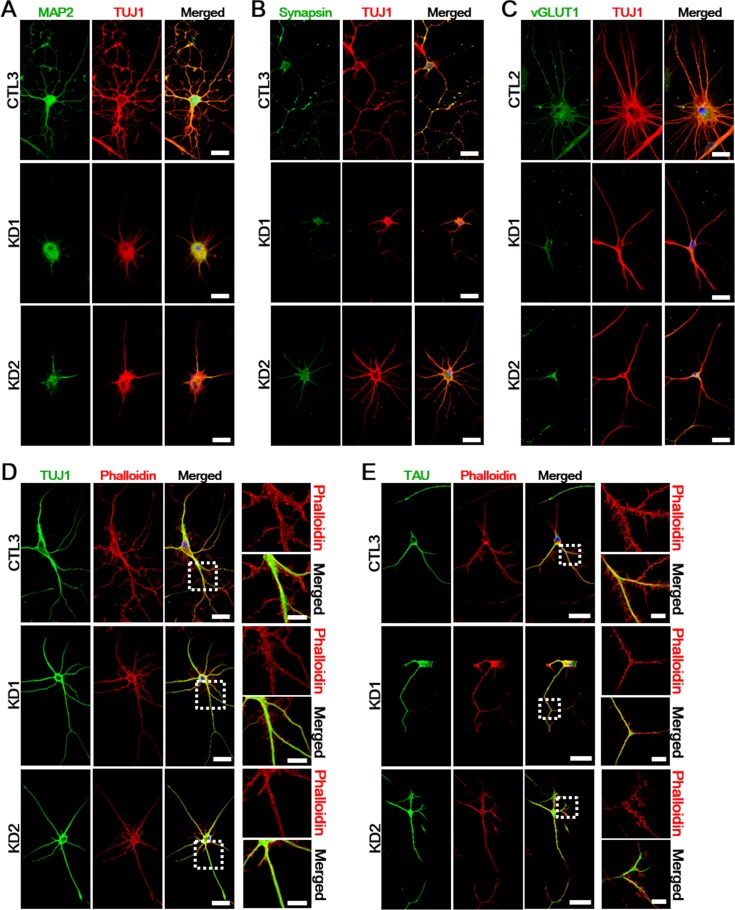
Defects of neuritic properties in KD iNeurons **A.**-**C.** Ten days after neuronal induction, expression of mature neuronal markers show reduced neurite outgrowth in KD iNeurons (*n* = 5). Red: TUJ1; green: MAP2 **A.**, Synapsin **B.**, vGLUT1 **C.**; blue: DAPI. Scale bars: 50 μm. **D.**-**E.** At twenty days after neuronal induction, we visualized actin filaments by phalloidin staining to investigate the cytoskeletal organization of a representative healthy control and KD iNeurons. The phalloidin double-labeled with TUJ1 **D.** and TAU **E.** showed abnormal neuritic branching and defective axonal outgrowth in both patients compared to the healthy controls. The zoomed insets of highlighted regions are presented to the right of each image (*n* = 3). DNA is identified by DAPI. Scale bars: 50 μm (enlarged images: 20 μm).

Axonal defects appear to directly contribute to the Twitcher pathogenesis in the murine model. Pathological axon swellings and early axonal loss have been shown to precede demyelination in isolated mutant neurons, thus suggesting an autonomous neuronal damage in the Twitcher mutants [7, 8]. To explore if genetic defects in KD patients affect neuronal or axonal pathology, we assessed myelin-independent neurite outgrowth and axonopathy on KD iNeurons in comparison with healthy controls. In line with previous studies on the Twitcher mouse model, iNeurons stained with TUJ1, MAP2, synapsin (neuronal marker), and vesicular glutamate transporter 1 (vGLUT1; glutamatergic neuronal marker) revealed that KD iNeurons, after culturing for 10 days *in vitro*, had rounded cell body with neurites shorter than those found in control iNeurons (Figure 5A-5C). In addition, we also noted the decreased number of neurites and axonal swellings in KD iNeurons compared to the healthy controls. In order to examine potential difference in cytoskeletal structure between controls and KD patient iNeurons, we stained the cells with fluorescent phalloidin for actin. The phalloidin double-labeled with early neuronal (Figure 5D) and axonal marker (Figure 5E) showed shorter and disorganized actin filaments with less neuritic branching and defective axonal outgrowth in both patients compared to controls. These data established a linkage of defective GALC to neuronal defects.

### Psychosine treatment causes neuropathology in control iNeurons

**Figure 6:**
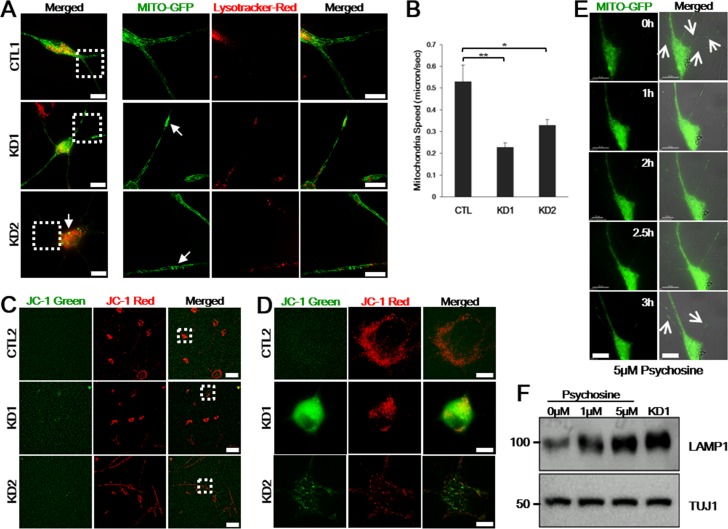
Psychosine induces lysosome accumulation, neurite and mitochondrial defects in control iNeurons **A.** Representative healthy control and KD iNeurons were transfected with Mito-GFP to visualize mitochondria and incubated with Lysotracker-Red to visualize lysosomes in live cells. The right panels represent zoomed images of the boxed areas on the left. Mito-GFP are distributed evenly throughout the dendrites in live control iNeurons, while those are swollen or accumulated in live KD iNeurons (arrows) (*n* = 7). Scale bars: 25 μm (zoomed images: 15 μm). **B.** Reduced moving speeds of mitochondria in patient iNeurons were measured using kymograph analysis. Moving speed is presented as means ± SEM, *n* = 80. One-way ANOVA followed by Tukey multiple comparisons test; *p < 0.05, **p < 0.001. **C.** JC-1 staining on live iNeurons from a representative control and the patients. Figure **D.** represents zoomed images of the boxed areas in **C.**. Control iNeurons have red fluorescence, sign of preserved mitochondrial membrane potential, whereas KD1 cells displayed strong green fluorescence and KD2 with weaker green fluorescence, sign of mitochondrial membrane depolarization (*n* = 5). Scale bars: 100 μm **C.**, 15 μm **D.**. **E.** Images of live control iNeurons expressing Mito-GFP after 5 μM of psychosine treatment. Representative frames from time-lapse image series (0-3 h) are shown. Neurites are abolished and mitochondria are accumulated (arrows). The right panel is merged pictures of DIC and fluorescence images. Scale bars: 25 μm. **F.** Immunoblotting detection of LAMP1 and TUJ1 neuronal marker confirmed that psychosine accumulation in healthy control iNeurons triggers lysosomal accumulation in a dose-dependent manner. LAMP1 of endogenous KD iNeurons is also presented.

During autophagy, autophagosome fuses with lysosome to degrade autophagy substrates. Because autophagic clearance of mitochondria is coordinated by lysosomes, aberrant lysosomes lead to mitochondrial dysfunctions, thus altering mitochondrial morphology [17, 18]. On the Twitcher model, both wild-type and mutant neurons exposed to psychosine showed defective mitochondrial transport, suggesting a causal cell autonomous effort of the metabolite [9]. To determine whether lysosomal alteration in KD patients also result in mitochondrial dysfunction, we compared mitochondrial morphology from KD patient-derived and control iNeurons by transfecting Mitochondria-targeted GFP (Mito-GFP) to label and observe mitochondria and by incubating with Lysotracker-Red to visualize lysosomes in live cells. Compared to control iNeurons, we found that both KD iNeurons clearly contained swollen, fragmented, or aggregated mitochondria (Figure 6A). Time-lapse movies recorded on the disease iNeurons demonstrated the induced morphological changes (Supplemental Movie 1). To analyze mitochondrial speed in iNeurons, we generated kymographs from these time-lapse movies using the kymograph plug-in of the ImageJ software. Reduction in the mean velocity of mitochondria was confirmed in both patient iNeurons (Figure 6B).

We also evaluated the mitochondrial membrane potential by using membrane-permeant dye tetraethlbenzimidzolylcarbocyanine iodide (JC-1). Cells with high mitochondrial membrane potential forms aggregate yielding red fluorescent whereas cells with low membrane potential forms monomeric form of JC1 yielding a diffuse green fluorescence. When we treated control iNeurons with valinomycin to induce mitochondrial matrix dissipation, the cells displayed green fluorescent signals, thus validating the assay (data not shown). We next loaded cultured iNeurons with JC-1 for 20 min and proceeded to live cell imaging. As shown in Figure 6C and 6D, the iNeurons derived from controls showed a strong red fluorescence without green fluorescence, while we observed a clear green fluorescence in KD1 and weaker green fluorescence in KD2 patient iNeurons indicative of impaired mitochondrial membrane potential in both patient iNeurons with a greater extent in iNeurons derived from the KD1 patient.

As GALC is responsible for catalyzing galactosylceramide (GalCer) and psychosine, defective GALC activity is expected to lead to the accumulation of psychosine to toxic levels, which has been demonstrated to be the cause for cell death in the Twitcher mouse model [19]. To determine whether psychosine is the cause for neuronal and axonal defects as shown in Figure 5 and mitochondrial defects characterized in Figure 6A-6D, we applied psychosine to Mito-GFP transfected control iNeurons. After 3 h of psychosine treatment, an increase in psychosine level caused both neurite defects and mitochondrial aggregation and swelling, thus recapitulating the typical phenotype observed in KD iNeurons (Figure 6E). To confirm the potential role of psychosine in causing neuronal death, we treated control iNeurons with different concentrations of psychosine. In a psychosine concentration-dependent fashion, control iNeurons demonstrated increased expression levels of LAMP1 as observed in KD iNeurons (Figure 6F). Together, these data demonstrated that KD iNeurons faithfully reflect the pathophysiology of KD patients, and more importantly, support the emerging hypothesis that psychosine accumulation in neurons triggers neuronal degeneration, which is likely a key event in the development of KD pathophysiology [7, 8, 20].

## DISCUSSION

ß-Galactosylceramidase (*GALC*) deficiency in Krabbe disease (KD) patients leads to the accumulation of its substrates galactosylceramide (GalCer) and psychosine [19, 21]. Since these defects have not been characterized on any cellular model of KD patients, we generated induced Neurons (iNeurons) from human skin fibroblasts from two adult-onset KD patients carrying heterozygous mutations in the *GALC* gene. One KD patient (KD1) carries one novel and one known mutation (p.[K563*];[L634S]) in its genome and the other KD patient (KD2) contains two known mutations but in a novel combination p.[N228_S232delinsTP];[G286D]. In order to establish iNeurons, we first confirmed a set of known disease pathologies in KD patient fibroblasts. The amount of lysosomes was increased and the morphology of lysosomes was altered with enlarged vesicles.

Importantly, recent studies on the Twitcher mouse model raised a new disease paradigm, suggesting an urgent need for understanding pathophysiological mechanisms of *GALC* mutations directly on relevant human models. Hence, we directly converted iNeurons from patient fibroblasts to demonstrate the molecular defects that became manifested only in KD iNeurons. Our study takes full advantage of an efficient trans-differentiation system, which does not involve intermediate pluripotent stem cells (iPSCs), to study such aspects in neuronal pathology associated with neurodegenerative diseases. The trans-differentiated cells demonstrated reduced GALC enzymatic activity, increased psychosine levels, axonal swelling and neurite defects in KD iNeurons, which are similar to the defects seen in the reported autopsy cases and in the Twitcher mouse model [7, 22].

In addition, we also detected morphological alterations in lysosomes and their accumulation in KD iNeurons. To elucidate whether the lysosomal alteration in KD patient iNeurons resulted in mitochondrial defects, we compared the morphology of mitochondria of live iNeurons derived from healthy controls and KD patients and observed irregularly shaped mitochondria and their fragmentation and aggregation in KD iNeurons. Since aberrant psychosine accumulation has been described as a cause of KD, we treated healthy control iNeurons with this metabolite and demonstrated the psychosine toxicity in a concentration-dependent manner as observed in KD iNeurons. Similarly, we recapitulated mitochondrial phenotypic defects in psychosine-treated control fibroblasts, supporting that lysosomal alterations result in mitochondrial defects [17]. Therefore, based on the results shown in this study, we conclude that, while fibroblasts may be a robust and feasible cell model, fibroblast-derived iNeurons are better for delineating KD pathologies.

As analysis of *GALC* genotypes could be easily performed on patient samples, previous studies on KD significantly correlated *GALC* genotypes with the KD disease phenotype. However, the correlation between mutant GALC activity and clinical severity has remained to be further substantiated, because deficient GALC activity in leukocytes apparently does not distinctly identify the clinical phenotypic characteristics [3, 4]. Distribution of a common mutation in adult-onset KD, GALC (p.L634S), for example, has been reported to be susceptible to a late-onset, mild form of KD [23]. However, the patients with the p.L634S mutation combined with other mutations or with the homozygous p.L634S mutation in the *GALC* gene exhibited a broad range of GALC activities, indicating that lower enzymatic activity does not always track the clinical severity [2–4]. Moreover, another common mutation in adult-onset KD GALC (p.G286D) exhibits a broad range of GALC activities when combined with another mutation or with the homozygous p.G286D mutation [4, 24], again indicating a limited correlation between the loss of enzymatic activity and clinical severity. Interestingly, GALC (p.G286D) was reported to directly cause slow progressive late-onset KD, whereas GALC (p. N228_S232delinsTP) was documented to be responsible for infantile-onset KD [2, 25, 26]. However, when the two mutations were combined in KD2 patient, the patient showed slow progression of the disease, which suggests limitations in studies identifying the disease exclusively based on the genotype and enzymatic activity measurements. To accurately link the loss of enzymatic activity and clinical severity, patient-specific disease modeling is clearly needed to elucidate the genotype-phenotype correlation. In this aspect, our current study suggests that patient-specific iNeurons carrying mutations in the *GALC* gene may serve as good cell models to recapitulate unique features of molecular defects associated with KD.

Recently, psychosine accumulation was shown to exist not only in myelinating glia, but also in KD neurons, suggesting the involvement of a myelin-independent neuronal pathogenesis [8, 9]. For modeling KD pathology, previous research relied on animal models to investigate the molecular mechanisms underlying KD pathogenesis. The Twitcher mouse carries a naturally occurring premature termination codon (p.W399*) in the *GALC* gene, resulting in a complete loss of the GALC enzymatic activity. Accordingly, this authentic model of KD is in keeping with the pathology observed in human infantile-onset KD, suggesting the pathological basis for KD in humans and offering a model for testing potential therapies against the disease, such as hematopoietic stem cell transplantation or enzyme replacement therapy [27]. Although the model has been used currently for defining pathogenesis, the Twitcher mouse model does appear to be fully adequate for studying the KD mechanism because the naturally occurring mutation in both alleles in *Galc* in the Twitcher mouse has never been found in KD patients. In this study, we present the first study towards understanding the KD pathology on a human iNeuron model and showed the direct link of KD to autonomous neuropathy. Using such patient-derived iNeurons, we not only found the accumulation of lysosomes, but also lysosomal and mitochondrial morphological defects. Intriguingly, treating control iNeuron with psychosine disrupted morphologies of both lysosomes and mitochondria and increased their aggregation in a concentration-dependent manner. These findings provide key evidence for the hypothesis that psychosine toxicity underlies neuronal dysfunction in KD patients [7].

In conclusion, this study explores human iNeurons as patient-specific cell models. Addressing the obscure correlation between GALC enzymatic activity and clinical phenotype due to the lack of an adequate disease model, we investigated neuropathology in KD patients by generating patient-specific iNeurons. Our findings report the defects in organelles and GALC substrates, resulting in autonomous neuronal KD pathology. Psychosine treatment of healthy control iNeurons confirmed the psychosine toxicity hypothesis. This experimental strategy is applicable to *GALC* genotypes associated with both infantile-onset and adult-onset KD. Moreover, the rapidly converted iNeurons from KD patient will enhance mechanistic studies of the disease and development of various treatment options on patient-specific iNeurons will expedite the development of effective therapeutics against KD.

## MATERIALS AND METHODS

### Genomic DNA analysis

The study protocol was approved by the Institutional Review Board of Hanyang University Hospital (IRB# 2011-R-63). Both patients and their family members were given written informed consent for direct sequencing of the *GALC* gene. Their genomic DNA were prepared from peripheral blood leukocytes using a Wizard Genomic DNA Purification kit (Promega). All 17 coding exons and flanking intronic regions of the *GALC* gene were amplified by polymerase chain reaction (PCR) using a set of specific primers. Direct sequencing was carried out using a BigDye Terminator Cycle Sequencing Ready Reaction kit (Applied Biosystems) on an ABI3100 Genetic Analyzer (Applied Biosystems). The characteristics of the KD and non-KD individuals whose samples were utilized for this study are summarized in Table 1.

### Cell cultures and conversion of human skin fibroblasts to iNeurons

HeLa cells were cultured in Dulbecco's modified Eagle's medium (DMEM) supplemented with 10 % fetal bovine serum (Gibco), sodium bicarbonate, sodium pyruvate (Sigma-Aldrich), and antibiotics. For transfection of HeLa cells, the cDNA encoding wild-type human *GALC* C-terminally tagged with FLAG was cloned into the pcDNA3.1 vector (invitrogen). Four *GALC* mutations (p.L634S, p.K563*, p.G286D, p.N228_S232delinsTP) with either FLAG or HA tags were introduced by PCR-based mutagenesis. The cells were transiently transfected with each wild-type or mutant constructs using Lipofectamine 2000 (Invitrogen) according to the manufacturer's instructions. The cells at 48 h post-transfection were fixed for immunostaining.

Fibroblasts were obtained from forearm skin with punch biopsy. Fibroblasts were cultured and maintained in DMEM supplemented with 20% FBS, non-essential amino acids (all from Gibco), sodium bicarbonate (Sigma), and 1% (vol/vol) Penicillin/Streptomycin/Fungizone (Cellgro). In all experiments, passage-matched fibroblasts (passages 3-5) were used. Fibroblasts were seeded at a density of 1×10^4^ cells/cm^2^ and used for experiments after cell synchronization by serum starvation at matched time points.

For direct conversion procedure, human fibroblasts were seeded onto matrigel (BD Biosciences)-coated 24-well tissue culture dishes or cell culture flasks (Nunc). Human iNeurons were generated from fibroblasts using lentiviral transduction of specific shRNAs against human PTBP1 (Sigma MISSION) according to the protocol described by Lim et al. [14]. Briefly, after the shRNA treatment, the cells were selected with 1 μg/ml puromycin. Selected cells were replaced for 3 days in N3 media (DMEM/F12 (Gibco) supplemented with 25 μg/ml Insulin, 50 μg/ml apo-transferrin, 20 nM progesterone, 100 nM putrescine, and 30 nM sodium selenite (all Sigma)), 10 ng/ml bFGF (Gibco), supplemented with BDNF, CNTF, GDNF, and NT3 (all PeproTech) as previously described [13, 14]. From day 4 to the day of analysis, the cells were maintained in N3 media supplemented with 2% FBS.

### Enzyme assays

Activity of β-galactosylceramidase in lymphocytes was determined by a high performance liquid chromatography tandem mass spectrometry LC-MS/MS, expressed as nmol/hr/mg protein. For enzymatic activity in iNeurons, 15 μg sonicated protein extract (50 μl) were mixed with 100 μl of 1.5 mM the fluorogenic substrate 4-methylumbelliferyl-β-D-galactoside (MUGal) (Sigma) resuspended in 0.1/0.2 M citrate/phosphate buffer, pH 4.0. Reactions were incubated 30 min at 37°C and then stopped with 0.2 M Glycine/NaOH, pH 10.6. Fluorescence of liberated 4-methylumbelliferone was monitored by absorbance at λex 360 nm and λem 446 nm and calculated the activity from a standard 4-methylumbelliferone (Sigma) curve [15].

### Mass spectrometry

For psychosine identification, lipid extractions from iNeurons were analyzed using a matrix-assisted laser desorption and ionization time-of-flight (MALDI-TOF) Axima Confidence™ spectrometer. For the lipid extraction from cells, 10 μl of lysates from iNeurons received 30 μl methanol and proceeded to centrifugation after 1 hr of vigorous shaking. Extracted lipids were then mixed with an equal quantity of α-cyano-4-hydroxycinnamic acid (CHCA) matrix and 1 μl of that mixture was loaded into the spectrometer sample plate. The samples were allowed to dry and were analyzed by MALDI-TOF mass spectrometry in positive ion mode. Psychosine is identified and quantified by mass/charge (m/z) in relation to standard psychosine (Santa Cruz Biotechnology).

### Immunostaining and confocal microscopy

Cells were washed with 1×PBS, fixed with 4% paraformaldehyde (PFA) for 15 min at room temperature, washed three more times with PBS, and immunofluorescence staining was performed. Cells were permeabilized with 0.3% Triton X-100 for 10 min at room temperature, and blocked for 1 hr in 5% normal goat serum (Vector Labs) after washing with PBS three times. Cells were incubated with primary antibodies for 2 hrs at room temperature, washed three times with 1×PBS, and detected with secondary antibodies for 1 hr at room temperature. For actin counterstaining, iNeurons were incubated with rhodamine-labelled phalloidin (Thermo Fisher Scientific, 1:400) for 20 min. After three additional washings with 1×PBS, nuclei were stained with DAPI. Coverslips were mounted on glass slides with Fluoromount-G (SouthernBiotech). Primary antibodies were mouse monoclonal antibodies against LAMP2 (monoclonal, Abcam, 1:1000), calnexin (Thermo Fisher Scientific, 1:100), and rabbit polyclonal antibodies to LAMP1 (monoclonal, Cell Signaling Technology, 1:1000). For neuronal cell markers, mouse monoclonal antibody against β-tubulin III (TUJ1; monoclonal, Covance, 1:1000) and rabbit polyclonal antibody to MAP2 (polyclonal, Cell Signaling Technology, 1:100), Synapsin I (polyclonal, Chemicon, 1:200), vGLUT1 (polyclonal, Synaptic Systems, 1:1000), and Tau (polyclonal, Thermo Fisher Scientific, 1:300) were used. Secondary antibodies were Alexa Fluor 488-conjugated and/or tetramethylrhodamine B isothiocyanate (TRITC)-conjugated mouse or rabbit antibodies (Gibco), and STAR 440SX-conjugated rabbit antibodies (Abberior) for stimulated emission depletion (STED) imaging. Images were acquired with a Leica TCS SP5 confocal microscope or using a Leica TCS SP8 STED microscope.

### Live imaging

Cultured iNeurons were grown in imaging dishes (Chamber slide Lab-Tek II 4; Fisher). To visualize mitochondria, the cells were transfected with a Mito-GFP plasmid two days before the analysis. To visualize the lysosomes in live cells, iNeurons were incubated with 250 nM Lysotracker-Red DND-99 dye (Invitrogen) in medium at 37°C for 30 min. After washing twice with PBS the medium was replaced with fresh medium. Images were taken one frame every 3 sec, for 3 min. Detection of mitochondrial membrane potential changes in live iNeurons were performed using membrane-permeant tetraethylbenzimidazolylcarbocyanine iodide (JC-1) staining kit (Sigma) according to manufacturer's instruction. To measure the speed of mitochondria, kymographs were generated and analyzed using ImageJ (Multiple Kymograph plugin). For psychosine toxicity test, cells were treated with psychosine and then imaged for 3 h, 2 frames every 30 min. Live imaging was acquired using a DeltaVision fluorescence microscopy system (Applied Precision) installed at the Hanyang Center for Research Facilities, Seoul.

### Western blot analysis

iNeurons were collected on day 15, washed with PBS, and lysed using a protein extraction solution (Intron Biotechnology) containing phosphate inhibitor (Calbiochem). Cell lysates were centrifuged and protein concentrations were determined. 15 μg of whole-cell protein extracts from each sample were separated by 7.5% or 10% sodium dodecyl sulfate polyacrylamide gel electrophoresis (SDS-PAGE) and transferred at 400 mA onto PVDF membranes (GE Healthcare). Membranes were blocked with 5% skim milk. The primary antibodies used were mouse monoclonal antibodies against TUJ1 (monoclonal, Covance, 1:1000) and rabbit polyclonal antibodies against GALC (polyclonal, Santa Cruz Biotechnology, 1:500), LAMP1 (monoclonal, Cell Signaling Technology, 1:1000), and GAPDH (polyclonal, Santa Cruz Biotechnology, 1:500). Membranes were developed using the Enhanced Luminescence kit (GenDepot). For psychosine toxicity test, control iNeurons were treated with control vehicle (DMSO) or 1, 5 μM psychosine (Sigma) for up to 3 h.

### Vesicle-size measurements

Confocal and STED images of fibroblasts or iNeurons were acquired after staining with anti-LAMP1 and random sections from cells were selected for analysis. The vesicles with morphologies with circular in geometry were chosen for measurements. Regions of interest (ROIs) were drawn over images and were measured and documented for statistical analysis. Clustered lysosomes were not included in the size analysis.

## SUPPLEMENTARY MATERIALS FIGURES AND TABLES




















