# Supplementary material for: Patient fibroblasts-derived induced neurons demonstrate autonomous neuronal defects in adult-onset Krabbe disease
Source: Oncotarget. 2016 Oct 21;7(46):74496–509. doi: 10.18632/oncotarget.12812 (PMC5342682; doi:10.18632/oncotarget.12812)
Supplement: Supplementary file 1 [file oncotarget-07-74496-s001.pdf]

Patient fibroblasts-derived induced neurons demonstrate autonomous neuronal defects in adult-onset Krabbe disease

Supplementary Material

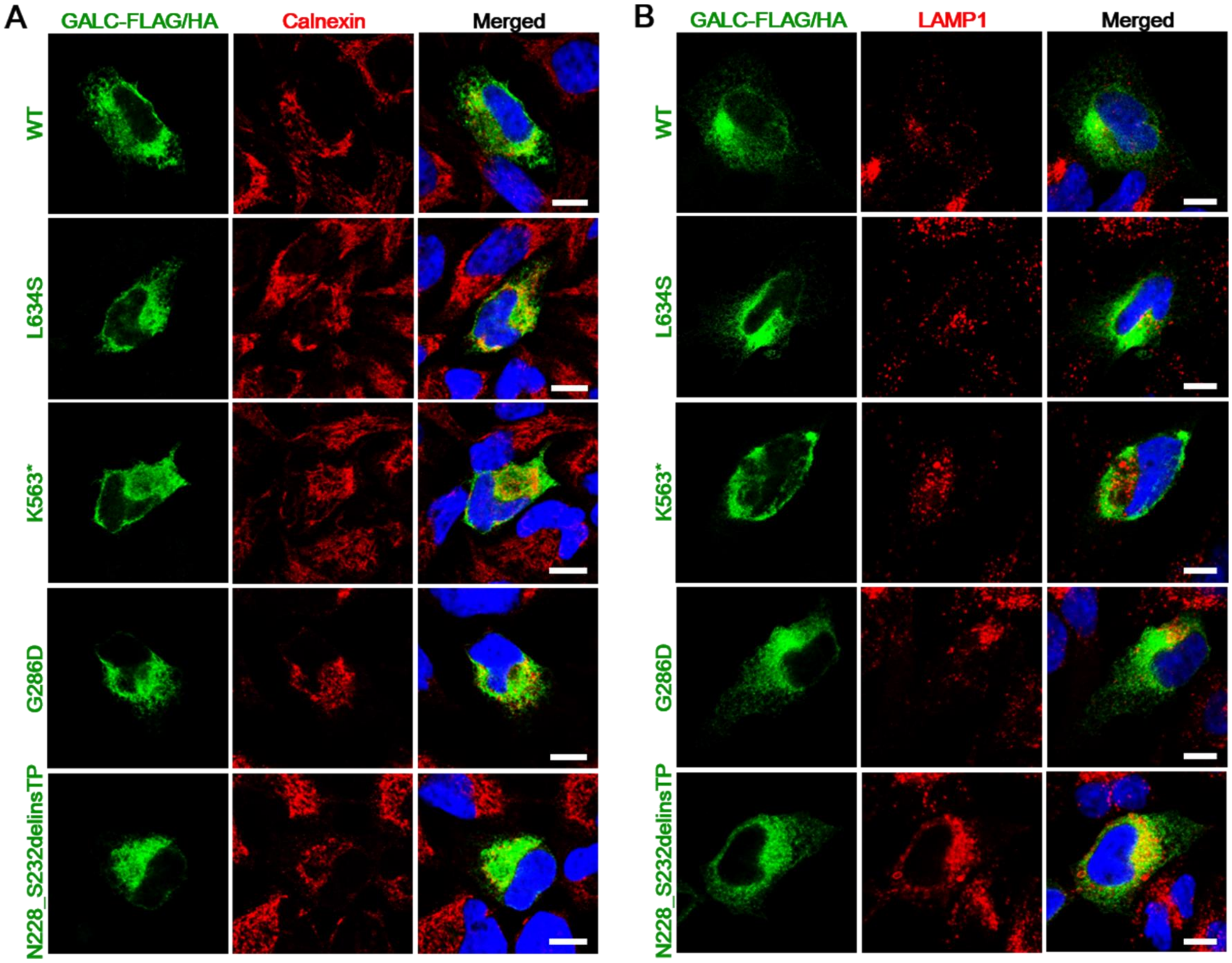

Supplemental Figure 1: **GALC distribution in transfected cells.** (A) Immunofluorescence for wild-type or the four mutant GALCs tagged with FLAG or HA in HeLa cells were stained with anti-calnexin or anti-LAMP1 antibodies. In all transfected mutant GALCs, the expression pattern is indistinguishable from that of the wild-type enzyme. GALCs were predominantly detectable in the ER. DNA is identified by DAPI. Scale bars: 10 μm.

Supplemental Movie 1: **Impairment of mitochondria in patient iNeurons.** (A) A representative control and two KD patient iNeurons were transfected with Mito-GFP to visualize mitochondria and incubated with Lysotracker-Red to visualize lysosomes in live cells. Mito-GFP are elongated string pattern throughout the dendrites in live healthy control iNeurons, while aberrant fragmentations or accumulations of mitochondria are observed in live patient iNeurons (B) KD1 and (C) KD2. Scale bars: 15 μm.
